# Origin and Reticulate Evolutionary Process of Wheatgrass *Elymus trachycaulus* (Triticeae: *Poaceae*)

**DOI:** 10.1371/journal.pone.0125417

**Published:** 2015-05-06

**Authors:** Hongwei Zuo, Panpan Wu, Dexiang Wu, Genlou Sun

**Affiliations:** 1 College of Agronomy, Anhui Agricultural University, Hefei, Anhui, China; 2 Biology Department, Saint Mary’s University, Halifax, Nova Scotia, Canada; Murdoch University, AUSTRALIA

## Abstract

To study origin and evolutionary dynamics of tetraploid *Elymus trachycaulus* that has been cytologically defined as containing **StH** genomes, thirteen accessions of *E*. *trachycaulus* were analyzed using two low-copy nuclear gene *Pepc* (phosphoenolpyruvate carboxylase) and *Rpb2* (the second largest subunit of RNA polymerase II), and one chloroplast region *trnL–trnF* (spacer between the tRNA Leu (UAA) gene and the tRNA-Phe (GAA) gene). Our chloroplast data indicated that *Pseudoroegneria* (**St** genome) was the maternal donor of *E*. *trachycaulus*. *Rpb2* data indicated that the **St** genome in *E*. *trachycaulus* was originated from either *P*. *strigosa*, *P*. *stipifolia*, *P*. *spicata* or *P*. *geniculate*. The *Hordeum* (**H** genome)-like sequences of *E*. *trachycaulus* are polyphyletic in the *Pepc* tree, suggesting that the **H** genome in *E*. *trachycaulus* was contributed by multiple sources, whether due to multiple origins or introgression resulting from subsequent hybridization. Failure to recovering **St** copy of *Pepc* sequence in most accessions of *E*. *trachycaulus* might be caused by genome convergent evolution in allopolyploids. Multiple copies of **H**-like *Pepc* sequence from each accession with relative large deletions and insertions might be caused by either instability of *Pepc* sequence in **H**- genome or incomplete concerted evolution. Our results highlighted complex evolutionary history of *E*. *trachycaulus*.

## Introduction

Interspecific or intergeneric hybridization and polyploidization are two widespread and evolutionarily important phenomena in plants, which play important roles in the formation of new allopolyploid species [1–3]. Numerous studies have indicated that many intra- and inter-genomic changes that accompanied allopolyploid formation such as rapid elimination and recombination of low-copy sequence fragment, DNA methylation pattern changes, retrotransposon activation, intergenomic conversion and epigenetic changes, might have produced a more harmonious behavior and activity of the different constituent genomes. More importantly, those genomic alterations exhibited different evolutionary dynamics which might lead to genetic asymmetry evolution resulting in conformity and convergent effects [4–9].

The tribe Triticeae combines a wide variety of biological mechanisms and genetic systems, and is an excellent group for studying evolutionary dynamics and speciation in plants [10]. Within this tribe, *Elymus* L. is the largest genus composed exclusively of allopolyploids with approximately 150 species [11]. Five basic genomes (**St**, **H**, **Y**, **P**, and **W**) have been cytologically assigned to the species in this genus (Genome symbols follow [12]). The **St** genome found in all *Elymus* species was supposedly donated by *Pseudoroegneria* (Nevski) Á. Löve. The **H**, **P**, and **W** genomes were derived from *Hordeum* L., *Agropyron* Gaertn., and *Australopyrum* (Tzvelev) Á. Löve, respectively, while the origin of **Y** genome is unknown [13–21].

*Elymus trachycaulus* (Link) (2n = 4x = 28) is a short-lived perennial, self-pollinating allotetraploid species. The distribution range of *E*. *trachycaulus* extends from Alaska to Newfoundland and all the way down south to Mexico, and usually grows in open forests and along roadsides [22]. The number of infraspecific taxa in the *E*. *trachycaulus* complex that are currently recognized varies from three to six, but considerably more have been recognized in the past [23]. The delimitation of taxa within *E*. *trachycaulus* complex is controversial and difficult, since the morphological characters used to distinguish infraspecific taxa (for instance, length and density of the spike), are at least partially under environmental control. Adding to this difficulty are some relatively distinct entities linked by morphologically intermediate plants derived from hybridization [23]. Previous studies have shown that *E*. *trachycaulus* complex is the most morphologically and geographically diverse species of *Elymus* in North America [24], and have showed considerable diversity [24–33]. Like most North American species of *Elymus*, *E*. *trachycaulus* is a tetraploid that combines the genomes of a *Pseudoroegneria* species (**St** genome) and a wild *Hordeum* species (**H** genome) [34–37], but little more is known about its origin and evolutionary dynamics.

In this study, we used two single copy nuclear genes: the second largest subunit of RNA polymerase II (*Rpb2*) and the phosphoenolpyruvate carboxylase (*Pepc*), along with chloroplast DNA *trnL–trnF* region (spacer between the tRNA-Leu (UAA) gene and the tRNA-Phe (GAA) gene) to explore genome evolutionary dynamics and the origin of tetraploid *E*. *trachycaulus*.

## Materials and Methods

### Plant materials and DNA extraction

Thirteen accessions of *E*. *trachycaulus* species were analyzed. DNA was extracted from fresh young leaf using the method of [38]. Two low copy nuclear gene (*Rpb2* and *Pepc*) and chloroplast *TrnL/F* sequences from different accessions of *E*. *trachycaulus* were amplified and sequenced. *Rpb2*, *Pepc* and *TrnL/F* sequences for some diploid Triticeae species representing the **St**, **H**, **I**, **Xa**, **Xu**, **W**, **P**, **E** and **V** genomes were obtained from the published data [39–41], and included in the analyses. Plant materials with accession number, genomic constitution, geographical origin, and GenBank identification number are presented in Table 1.

**Table 1:** Taxa from *Bromus*, *Aegilops*, *Eremopyrum*, *Heteranthelium*, *Psathyrostachys*, *Secale*, *Taeniatherum*, *Agropyron*, *Australopyrum*, *Dasypyrum*, *Thinopyrum*, *Triticum*, *Pseudoroegneria*, *Hordeum* and *Elymus* used in this study, their origin, accession number and GenBank sequence number.

Species	Accession No.	Genome	Origin	*RPB2*	*PepC*	*TrnL/F*
*B*. *tectorum* L.	Kellogg s.n.		NA	-	AY553239	-
*B*. *tectorum* L.			NA	-	-	AB732928
*B*.*catharticus* Vahl	CN32048		NA	HQ014410	-	-
*B*. *inermis* Leyss.	PI618974		Xinjiang, China	GQ848517	-	-
*Aegilops comosa* Sibth. and Smith	G 602	M	NA	-	AY553236	-
*Aegilops sharonensis* Eig.	PI584396	S^1^	Israel			EU013659
*Aegilops speltoides* Tausch	Morrison s.n.	S	NA	-	-	AF519112
*Eremopyrum triticeum* (Gaertn.) Nevski		F	NA	KC545625	-	-
*Eremopyrum bonaepartis* (Spreng.) Nevski	H5554	F	NA	-	-	AF519148
*Eremopyrum orientale* (L.) Jaub. & Spach	H 5555	F	NA	-	AY553254	AF519151
*Heteranthelium piliferum* (Banks & Sol.) Hochst.	PI 402352	Q	Iran	-	AY553255	AF519153
*Eremopyrum distans* (K. Koch) Nevski		F		KC545624	-	-
*Psathyrostachys juncea* (Fischer) Nevski	PI206684	Ns	Turkey	-	-	AF519170
*Psathyrostachys huashanica* Keng ex Kuo				KC545696	-	-
*Psathyrostachys lanuginosa* (Trin.) Nevski				KC545697	-	-
*Secale cereale* L.	Kellogg s.n.	R	NA	-	-	AF519162
*Taeniatherum caput-medusae* (L.) Nevski	RJMG 189	Ta	NA	-	AY553268	-
	MB-106-41-79	Ta	NA	-	-	AF519164
*Ag*. *cristatum* (L.) Gaertn.	PI 383534	P	Kars, Turkey	EU187438	-	-
	PI 279802	P	Ontario, Canada	KC545622-	AY553237	-
*Ag*. *mongolicum* Keng	D2774	P	NA	-	-	AF519117
*Aust*. *retrofractum* (Vickery) Á. Löve	Crane 86146	W	NA	-	-	AF519118
*Aust*. *velutinum* (Nees) B. K. Smion	D 2873–2878	W	NA	-	AY553238	AF519119
*D*. *villosum* (L.) P. Candargy	PI 368886	V	Gaziemir, Turkey	EU187471	-	-
	D 2990	V	NA	-	AY553240	-
*Douglasdeweya deweyi* (Jensen, Halch & Wipff) C. Yen, J.L. Yang & B.R. Baum	PI531756	StP	NA	GQ867871	-	-
*Dasypyrum villosum* (L.) Candargy	PI251478	V	Turkey	-	-	AF519128
*Thinopyrum elongatum* (Host) D.R.Dewey		E^e^		KC545671	-	-
*Thinopyrum elongatum* (Host) D.R.Dewey	PI 142012	E^e^	Russia Federation	EU187439	-	-
*Thinopyrum elongatum*(Host) D.R.Dewey	RJMG 113	E^e^	NA	-	AY553269	-
*Thinopyrum elongatum* (Host) D.R.Dewey	PI531719	E^e^	France	-	-	AF519166
*Thinopyrum bessarabicum* (Savul. & Rayss) Á.Löve	PI531711	Eb	Estonia	-	-	AF519165
*Lophopyrum nodosum* (Nevski) Á. Löve	PI547344	StE	Kars, Turkey	GQ867867	-	-
*Lophopyrum caespitosum* (K. Koch) Á. Löve	PI547311	StE	Leningrad, Russian Federation	GQ867865	-	-
				GQ867866	-	-
*H*. *vulgare* L.	RJMG 107	I	NA	-	AY553260	-
*H*. *vulgare* L.	HT025	I		-	-	AJ969295
*H*. *spontaneum* K. Koch	HT025	I		-	-	AJ969296
*H*. *bulbosum* L.	PI 440417	I	NA	-	EU282294	AF519122
		I		-	EU282295	-
*H*. *marinum* Huds	PI 304346	Xa	California, USA	-	-	AF519124
*H*. *marinum* Huds		Xa		-	-	KF600707
*H*. *marinum* subsp. *gussoneanum* (Parlatore) Thellung		Xa		-	-	AB732935
*H*. *murinum* L.	PI 247054	Xu	California, USA	-	-	AF519125
*H*. *murinum* L.	CIho 15683	Xu	Oregon, USA	-	-	AF519126
*H*. *muticum* J. Presl	HT043	H		-	-	AJ969330
	PI 531791	H	NA	-	EU282302	-
*H*. *pusillum* Nutt.		H		-	-	AB732937
	CIho 15654	H	NA	-	EU282301	-
*H*. *stenostachys* Godr.	H 6439	H	Argentina	EU187473	-	-
*H*. *flexuosum* Nees ex Steud.	HT046	H	NA	-	-	AJ969333
*H*. *comosum* J. Presl	HT060	H	NA	-	-	AJ969362
*H*. *pubiflorum* Hook. f	HT075	H	NA	-	-	FM163499
*H*. *bogdanii* Wilensky	PI 531760	H	Xinjiang, China	-	EU282293	-
	PI531761	H	China	-	-	AY740789
*H*. *brevisubulatum* (Trin.) Link		H		KC545626	-	-
*H*. *roshevitzii* Bowden	HT005	H	NA	-	-	AJ969271
	PI 531781	H	NA	GQ848518	EU282297	-
*H*. *chilense* Roem. and Schult.	HT053	H	NA	-	-	AJ969351
*H*. *patagonicum* (Haumann) Covas	HT046	H	NA	-	-	AJ969336
*H*. *brachyantherum* subsp. *californicum* (Covas & Stebbins) Bothmer et al.		H	NA	-	-	KF600706
*H*. *brachyantherum* Nevski		H	NA	-	-	AJ969314
*P*. *geniculata* (Trin.) Á. Löve	PI565009	St	Russian Federation	GQ867874	-	-
*P*. *geniculata* subsp. *scythica* (Nevski) Á. Löve	PI502271	StE	Russian Federation	GQ867869	-	-
		StE		GQ867870	-	-
*P*. *kosaninii* (Nabelek) Á. Löve	PI237636	??	Turkey	GU073306	-	-
*P*. *libanotica* (Hack.) D. R. Dewey	PI 228389	St	Iran	HQ231837	-	AY730567
	PI 228391	St	Iran	-	EU282304	AF519156
	PI330687	St	Iran	EF596753	-	-
	PI 282392	St	Iran	-	EU282305	-
*P*. *spicata* (Pursh) Á. Löve	PI 506274	St	Washington, United States	EF596746	-	-
	PI 610986	St	Utah, United States	EF596747	AY553263	AF519158
	D 2844	St	NA	-	AY553264	-
	PI547161	St	Oregon, USA	-	-	AF519159
*P*. *spicata*	PI236681	St	Canada	-	-	AF519157
	D2839	St	NA	-	-	AF519160
*P*. *stipifolia* (Czern. ex Nevski) Á. Löve	PI 325181	St	Stavropol, Russian Federation	EF596748	-	-
	PI 313960	St	Russian Federation	-	EU282306	-
	PI 440095	St	Russian Federation	+	-	EU617255
*P*. *stipifolia* (Czern. ex Nevski) Á. Löve	PI 531751	St	NA	-	EU282307	EU617251
				-	EU282308	-
	PI636641	St	Krym, Ukraine	-	-	EU617252
*P*. *strigosa* (M. Bieb.) Á. Löve	PI 531752	St	Estonia	HQ231850	-	EU617284
	W6 14049	St	Russian Federation	HQ231836	-	-
	PI 499637	St	China	GQ848520	EU282309	EU617269
				-	EU282310	-
	PI531752	St	Estonia	GQ867876	-	-
				GQ867875	-	-
	PI531753	St	Estonia	KC545698	-	EU617283
*P*. *strigosa* subsp. *aegilopoides* (Drobov) Á.Löve	MA-109-31-50	St	NA	-	-	AF519155
	PI565082	St	Xinjiang, China	-	-	EU617262
	W6 13089	St	Xinjiang, China	HQ231835	-	EU617265
	PI 531755	St	China	-	EU282311	KF624612
	PI595164	St	China	-	-	EU617267
*P*. *gracillima* (Nevski) Á.Löve	PI 440000	St	Stavropol, Russian Federation	-	-	EU617289
*P*. *tauri* (Boiss. & Balansa) Á. Löve	PI 380652	St	Iran	-	EU282312	EU617312
	PI 401319	St	Iran	-	EU282313	-
	PI 380644	St	Iran	-	EU282314	-
				-	EU282315	-
	PI401320	St	Iran	-	-	EU617308
	PI401323	St	Iran	-	-	EU617305
*E*. *trachycaulus* (Link) Gould ex Shinners	PI537323	StH	Utah, United States	EU187479	-	-
*E*. *trachycaulus* (Link) Gould ex Shinners	H3526	StH	Nerungri, Russia	EF596764	-	-
*Elymus trachycaulus* (Link) Gould ex Shinners	PI372500	StH	Northwest Territory, Canada	-	-	AF519141
*Elymus trachycaulus* (Link) Gould ex Shinners	PI452446	StH	Canada	-	-	AF519142
*Elymus trachycaulus* (Link) Gould ex Shinners	PI372644	StH	Alaska, USA	KR063083	KR063054, KR063066	KR063050
*Elymus trachycauls* (Link) Gould ex Shinners	PI387895	StH	Beaverlodge, Alberta, Canada	KR063089, KR063100	KR063065, KR063077	KR063042
*Elymus trachycaulus* (Link) Gould ex Shinners	PI440098	StH	Tselinograd, Kazakhstan	KR063093,KR063079	KR063067, KR063070	KR063047
*Elymus trachycaulus* (Link) Gould ex Shinners	PI440101	StH	Shorthandy, Kazakhstan	KR063080, KR063098	KR063059, KR063072	KR063052
*Elymus trachycaulus* (Link) Gould ex Shinners	H10665A	StH	USA	KR063088	KR063068, KR063078	KR063044
*Elymus trachycaulus* (Link) Gould ex Shinners	H3526	StH	Nerungri, Russia	KR063081, KR063101	KR063057, KR063063	KR063045
*Elymus trachycaulus* (Link) Gould ex Shinners	H10140	StH	Altai, Russian Federation	KR063090, KR063094	KR063064	KR063040
*E*. *trachycaulus* subsp. *subsecundus* (Link) A.& D. Löve	PI232147	StH	USA	KR063091, KR063102, KR063104	KR063056, KR063062, KR063075	KR063049
*E*. *trachycaulus* subsp. *subsecundus* (Link) A.& D. Löve	PI232150	StH	USA	KR063082, KR063097	KR063073	KR063041
*E*. *trachycaulus* subsp. *subsecundus* (Link) A.& D. Löve	PI232151	StH	USA	KR063086	KR063053, KR063060	KR063043
*Elymus trachycaulus* (Link) Gould ex Shinners	H4228	StH	Lincoln County, Utah, USA	KR063085, KR063096	, KR063069	KR063046
*E*. *trachycaulus* subsp. *subsecundus* (Link) A.& D. Löve	PI236685	StH	Canada	KR063092, KR063103	KR063055, KR063058	KR063051
*Elymus trachycaulus* (Link) Gould ex Shinners	H3995	StH	Rich County,Utah, USA	KR063087, KR063099	KR063061, KR063071	KR063048

NA: information not available; +: sequence present,-: sequence absent

### DNA amplification and sequencing

The sequences of *Rpb2*, *Pepc* and cpDNA *TrnL/F* were amplified by polymerase chain reaction (PCR) using the primers P6F and P6FR [42], PEPC-F and PEPC-R [40], and TrnL and TrnF [41], respectively. DNA was amplified in a 20 μl reaction containing 20 ng template DNA, 0.25 mM of each deoxynucleotide (dATP, dCTP, dGTP and dTTP), 2.0 mM MgCl2, 2.0 U *Taq* polymerase (TransGen, Beijing, China), 0.25 μM of each primer. Amplification was performed in a DNA Thermo-cycler (iCycler, Bio-Rad). The amplification profile for the *Rpb2* gene was: an initial denaturation at 94°C for 4 min; 35–40 cycles of 94 C for 40 s, 60°C for 50 s, 72°C for 2 min, and a final cycle of 72°C for 10 min. The PCR profile for amplifying *Pepc* gene was: an initial denaturation at 94°C for 4 min; 38 cycles of 94°C for 40 s, 65°C for 50 s, 72°C for 2 min, and a final cycle of 72°C for 10 min. The PCR condition for *TrnL/F* was: 5 min at 95°C, 40 cycles of 30 s at 94°C, 1 min at 61°C, 2 min at 72°C, followed by 10 min at 72°C. PCR products were purified using the EasyPure Quick Gel Extraction Kit (TransGen, Beijing, China) according to manufacturer’s instructions. The PCR products of the nuclear genes amplified from *E*. *trachycaulus* were cloned into the pGEM-easy T vector (Promega Corporation, Madison, Wis., USA) according to the manufacturer’s instructions, and transformed into *E*. *coli* competent cell DH5α (TransGen, Beijing, China). 12–24 clones from each accession were sequenced. The PCR product amplified by cpDNA primer *TrnL/F* was purified and directly sequenced. Both the PCR products and positive colonies were commercially sequenced by the Shanghai Sangon Biological Engineering & Technology Service Ltd (Shanghai, China). To enhance the sequence quality, both forward and reverse strands were sequenced independently. To avoid any error which would be induced by *Taq* DNA polymerase during PCR amplification, each PCR product amplified by cpDNA primer *TrnL/F* was independently amplified twice and sequenced, since *Taq* errors that cause substitutions are mainly random and it is unlikely that any two sequences would share identical *Taq* errors to create a false synapomorphy.

### Data analysis

The chromatographs of automated sequence results were visually checked. Multiple sequence alignments were made using Clustal X with default parameters and additional manual editing to minimize gaps [43]. Maximum-parsimony (MP) analysis was performed using the computer program PAUP ver. 4 beta 10 [44]. All characters were specified as unweighted and unordered, and gaps were excluded in the analysis. Most-parsimonious trees were obtained by performing a heuristic search using the Tree Bisection-Reconnection (TBR) option with MulTrees on, and ten replications of random addition sequences with the stepwise addition option. Multiple parsimonious trees were used to generate a strict consensus tree. Overall character congruence was estimated by the consistency index (CI), and the retention index (RI). In order to infer the robustness of clades, bootstrap values with 1000 replications [45] were calculated. In addition to MP analysis, Bayesian analyses analysis was also performed. Eight evolution models of sequence were tested using PhyML 3.0 [46]. For each data set, the general time-reversible (GTR) [47] model led to a largest ML score compared to the other 7 substitution models: JC69 [48], K80 [49], F81 [50], F84 [51], HKY85 [52], TN93 [53] and custom (data not shown). As the result, the GTR model was used in the Bayesian analysis using MrBayes 3.1 [54]. MrBayes 3.1 was run with the program’s standard setting of two analyses in parallel, each with four chains, and estimates convergence of results by calculating standard deviation of split frequencies between analyses. In order to make the standard deviation of split frequencies fall below 0.01 so that the occurrence of convergence could be certain, 1,159,000 generations for *Rpb2* data, 1,037,000 generations for *Pepc*, and 4,110,000 generations for *TrnL/F* were run. Samples were taken every 1000 generations under the GTR model with gamma-distributed rate variation across sites and a proportion of invariable sites. For all analyses, the first 25% of samples from each run were discarded as burn-in to ensure the stationarity of the chains. Bayesian posterior probability (PP) values were obtained from a majority rule consensus tree generated from the remaining sampled trees.

## Results

### *Rpb2* analysis

Maximum parsimony analysis of 58 *Rpb2* sequences was conducted using *B*. *inermis* and *B*. *catharticus* as outgroup (125 parsimony informative characters, 316 equally most parsimonious trees, CI = 0.791; RI = 0.932).

**Fig 1:**
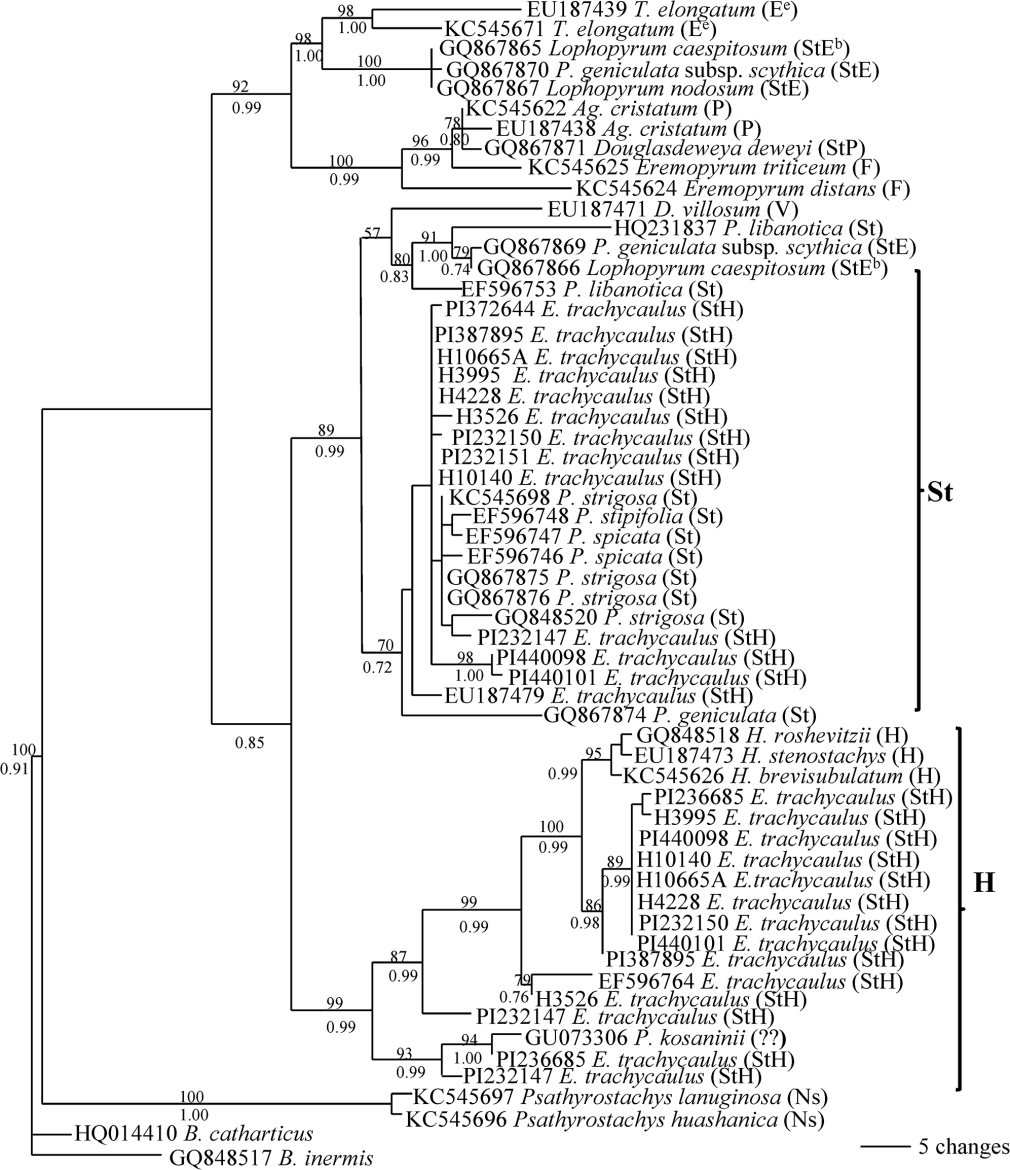
One of the 316 parsimonious trees derived from rpb2 sequence data was conducted using heuristic search with TBR branch swapping. Numbers above and below branches are bootstrap values from MP and Bayesian posterior probability (PP) values, respectively. *Bromus inermis* was used as an outgroup. Consistency index (CI) = 0.791, retention index (RI) = 0.932.

The separated Bayesian analyses using GTR model resulted in identical trees with mean log-likelihood values -3928.47 and -3973.80 (data not shown). The tree topology generated by Bayesian analyses using the GTR model is similar to those generated by maximum parsimony. One of the most parsimonious trees with Bayesian PP and maximum parsimony bootstrap (1000 replicates) value is shown (Fig 1).

Two distinct copies of sequences were recovered from each nine accessions of *E*. *trachycaulus* (PI387895, PI440098, PI440101, H10665A, H3526, H10140, PI232150, H4228, H3995). Phylogenetic analysis clearly separated the two copies of sequences from each accession into two distinct groups, one copy with **St** genome diploid species, and another copy associated with **H** genome diploid species. Two copies of sequences from accession PI 236685 were recovered, but both were grouped into **H** clade in 99% bootsrtap support and 0.99 PP (Fig 1). Three distinct copies of sequences were found from accession PI232147, one was grouped into the **St** and two were placed into the **H** genome clade. Only one copy of sequence from accession PI232151 was recovered, and was placed into the **St** clade. The three **H** genome species (*H*. *roshevitzii*, *H*. *stenostachys*, *H*. *brevisubulatum*) were grouped together with a 95% bootstrap support in MP, and 0.99 PP in Bayesian analysis, and was sister to the **H**-like copy sequences from nine accessions of *E*. *trachycaulus* which were grouped together in a 86% bootstrap support and 0.98 PP. The **H**-like sequence from EF596764 and H3526 formed a group, and was sister to the **H** genome diploids.

### *Pepc* analysis

**Fig 2:**
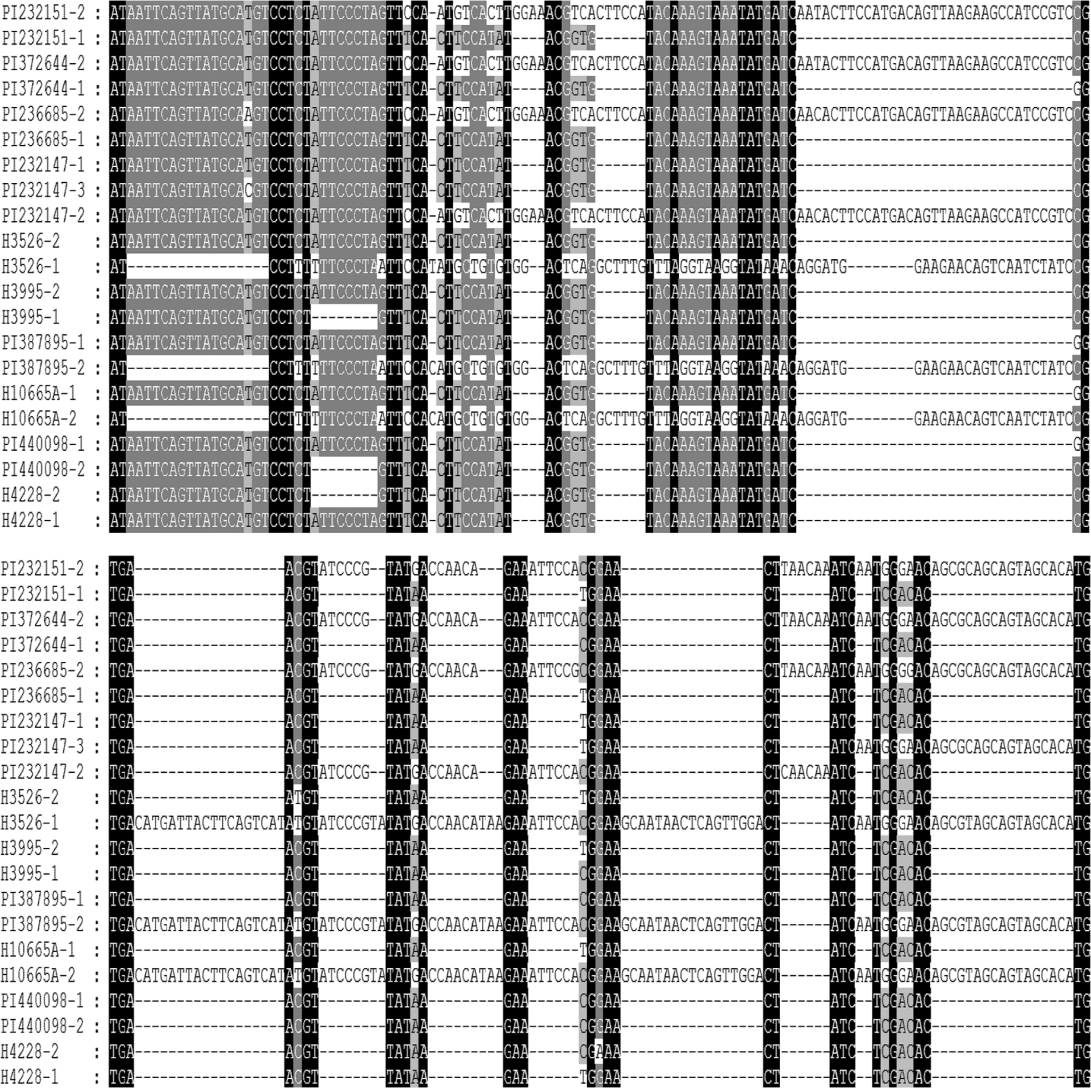
Multiple copies of the pepc sequences recovered from H genome of *Elymus trachycaulus* with relative large insertion/deletion, which might be caused by gene instabilities.

*Pepc* gene from 13 accessions of *E*. *trachycaulus* were cloned and sequenced. At least 10 clones from each cloned PCR product were screened and sequenced. Two distinct copies of sequences were recovered from each 9 accessions of *E*. *trachycaulus*. The relative large insertion/deletion was observed between the two copies sequences from each accession, and shown in Fig 2. Three copies of sequences were recovered from accession PI232147, and relative large insertion/deletion among the three copies of sequences was also observed (Fig 2). Only one copy of sequence was recovered each from accession H10140, PI 232150 and PI 440101.

**Fig 3:**
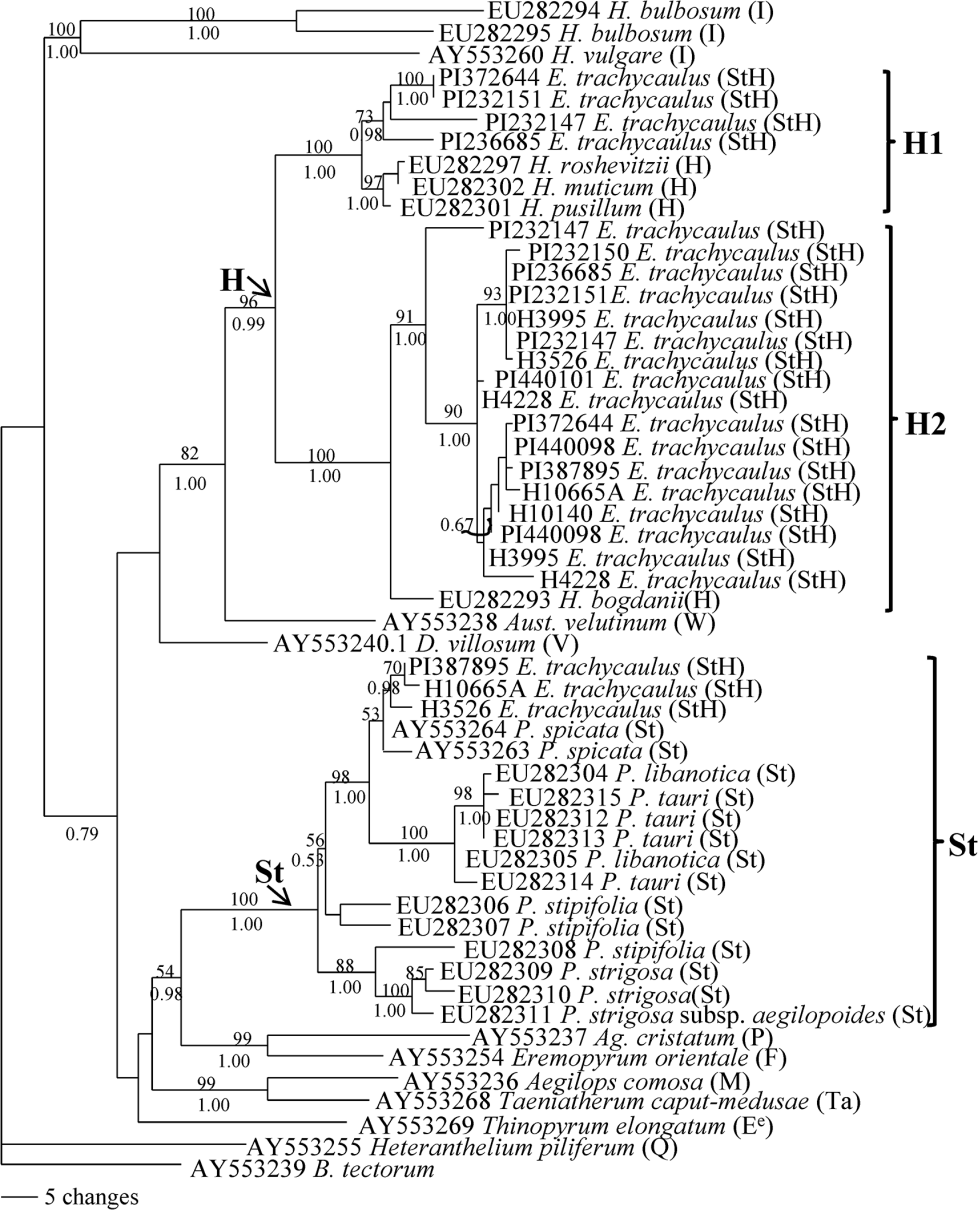
One of the 570 parsimonious trees derived from *pepc* sequence data was conducted using heuristic search with TBR branch swapping. Numbers above and below branches are bootstrap values from MP and Bayesian posterior probability (PP) values, respectively. *Bromus tectorum* was used as an outgroup. Consistency index (CI) = 0.735, retention index (RI) = 0.906.

Phylogenetic analysis of the 54 *Pepc* sequences was performed using *B*. *tectorum* as an outgroup. The data matrix contained 958 characters, of which 600 were constant, 154 were parsimony uninformative, and 204 were parsimony informative. Heuristic searches resulted in 570 most parsimonious trees with a CI = 0.735 (excluding uninformative characters), and RI = 0.906. The Bayesian analyses using GTR model resulted in identical trees with mean log-likelihood values -5561.85 and -5701.88 (data not shown). The tree topologies generated by MP and Bayesian analyses were similar to each other. One of the most parsimonious trees with BS and PP values is shown in Fig 3.

Phylogenetic analysis separated two copies sequences each from accession PI 387895, H10665a and H3526 into two distinct clades, one in **H** genome and another in **St** genome clade (Fig 3). However, the two different copies of sequences each from accession PI 232151, PI 372644, PI 236685, PI H3995, PI 440098 and H4228 were placed in the **H** clade with diploid *Hordeum* species together with a 96% bootstrap support and 0.99 PP. Within the **H** clade, two well separated subclades were observed. One contained 4 sequences from *E*. *trachycaulus* and the sequences from *H*. *roshevitzii*, *H*. *muticum* and *H*. *pusillum* in 100% BS and 1.00 PP support. Another contained 17 *E*. *trachycaulus* sequences and one *H*. *bodganii* sequence in 100% BS and 1.00PP support. Two different copies of sequences each from accession PI 372644, PI232151 and PI 236685 were separated into two **H** subclades, whereas two different copies of sequences each from accession PI 440098, H3995 and H4228 were placed into the same subclade (**H2**). Three different copies of sequences from accession PI 232147 were placed into the **H1**, **H2** and **St** clade, respectively.

### *TrnL/F* analysis

**Fig 4:**
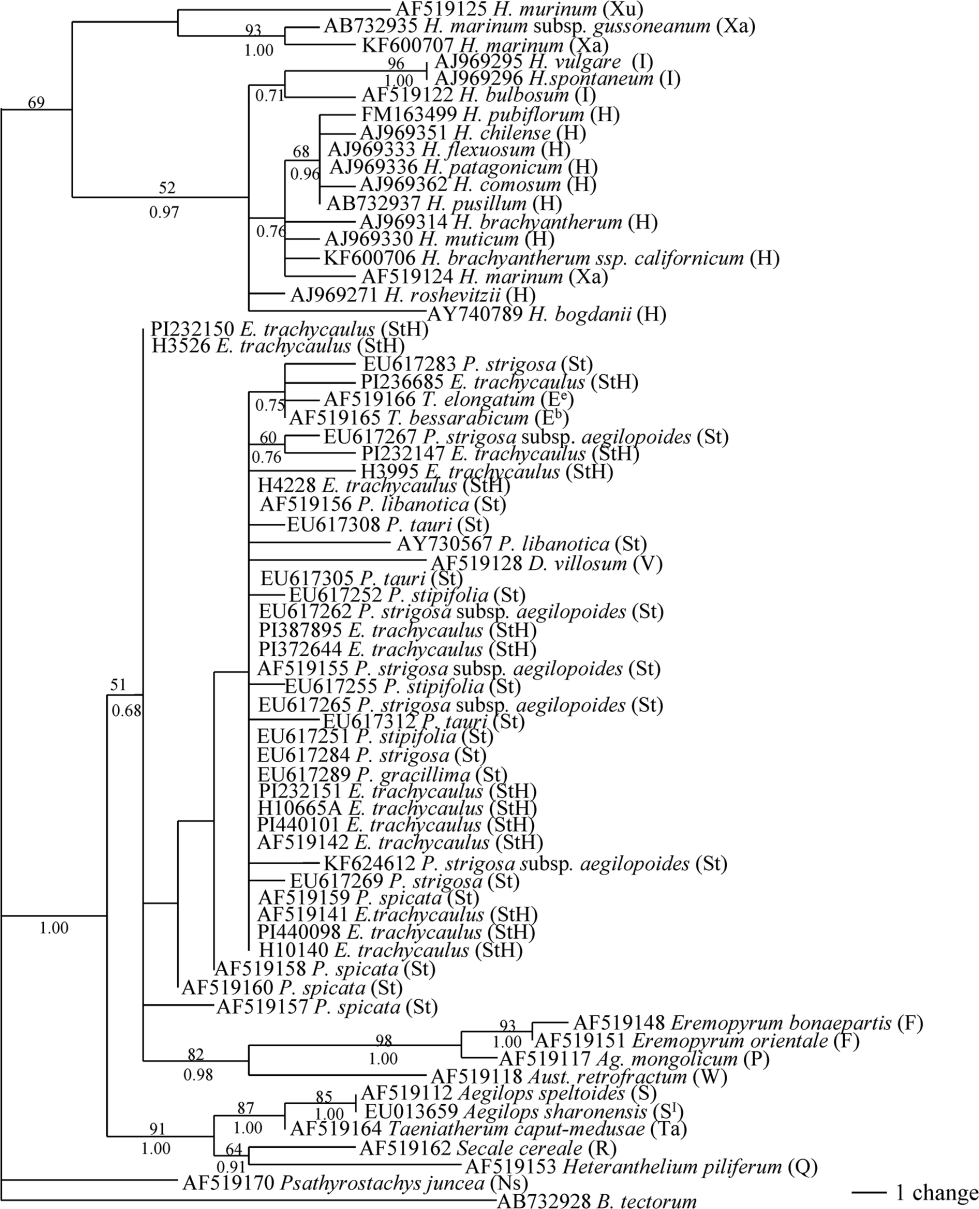
One of the 134 parsimonious trees derived from *TrnL/F* sequence data was conducted using heuristic search with TBR branch swapping. Numbers above branches are MP bootstrap values and Bayesian posterior probability (PP) values, respectively. *Bromus tectorum* was used as an outgroup. Consistency index (CI) = 0.903, retention index (RI) = 0.941.

Phylogenetic analysis of 67 *TrnL/F* sequences was performed using *B*. *tectorum* as an outgroup. The data matrix contained 793 characters, of which 684 were constant, and 36 were parsimony informative. Heuristic searches resulted in 134 most parsimonious trees with a CI = 0.903 (excluding uninformative characters) and RI = 0.941. The separated Bayesian analyses using GTR model resulted in identical trees with mean log-likelihood values -2494.43 and -2590.54 (data not shown). One of the most parsimonious trees with BS values from MP and PP value from Bayesian analysis is shown in Fig 4.

Phylogenetic analyses divided the 67 sequences into two clades. All sequences from *Hordeum* species were placed into one clade with 69% bootstrap support. All sequences from *E*. *trachycaulus* were grouped with the **St** genome, **E** ^**b**^, **E** ^**e**^, **V**, **W**, **P** and **F** in a 51% bootstrap value and 0.68 PP. Within this clade, the sequence from accession PI232147of *E*. *trachycaulus* formed a subclade with *P*. *strigosa* subsp. *aegilopoides* (**St**) in 60% SB and 0.76 PP support. The sequences from **F**, **P** and **W** genomic species formed a subclade in 82% BS and 0.98 PP support.

## Discussion

### Multiple origins of *E*. *trachycaulus*

Previous studies using cpDNA sequences have confirmed that the diploid **St** genome species, *Pseudoroegneria*, is the maternal donor of *E*. *trachycaulus* [41, 54–56]. At present study, the phylogenetic analysis of *TrnL/F* data placed all sequences from *E*. *trachycaulus* with the sequences from *Pseudoroegneria* (**St**), *Thinopyrum* (**E** ^**b**^, **E** ^**e**^), *Dasypyrum* (**V**), *Agropyron* (**P**), *Eremopyrum* (**F**) and *Australopyrum* (**W**) (Fig 4). It is difficult to rule out *Thinopyrum*, *Dasypyrum*, *Agropyron*, *Eremopyrum* and *Australopyrum* as potential maternal donors to *E*. *trachycaulus*. Our result is consistent with a study based on combined cpDNA restriction sites, rpoA sequences, and tRNA spacer sequences, in which the several North American *Elymus* species including *E*. *trachycaulus* were also grouped with *Pseudoroegneria*, *Thinopyrum* and *Dasypyrum* [41]. In contrast to the chloroplast *TrnL/F* data, phylogenies of two nuclear gene sequences (*Rpb2* and *Pepc*) placed the *E*. *trachycaulus* into *Pseudoroegneria* and *Hordeum* clades, and clearly separated from the *Thinopyrum*, *Dasypyrum* and other diploid species analyzed here (Figs 1 and 3). Thus, *Pseudoroegneria* as a maternal donor to *E*. *trachycaulus* is consistent with nuclear data in this study and previous chloroplast data [41, 54–56], as well as genome-pairing data [13]. Two distinct copies of *Rpb2* sequences each from 9 out of thirteen accessions of *E*. *trachycaulus* were obtained, and were separated into **St** and **H** clades by phylogenetic analysis, indicating that the **StH** genome constitution of these nine accessions (PI387895, PI440098, PI440101, H10665A, H3526, H10140, PI232150, H4228, H3995) of *E*. *trachycaulus*. The *Pepc* sequence data confirmed the presence of **StH** genome in PI 387895, H10665A and H3526 (Fig 3). Sequence alignment revealed two distinct copies from each accession PI440098, H4228, PI 236685 and H3995. However, phylogenetic analysis grouped the two copies sequences each from accession PI440098, H4228, and H3995 into the **H2** group, while the two copies of sequences from accession PI236685 were separated into **H1** and **H2** groups (Fig 3). Only one copy of *Pepc* sequence each was recovered from accession PI 440101, H10140 and PI 232150, and grouped into **H2** group. Both *Rpb2* and *Pepc* data suggested that accession PI 236685 contained two different copies of **H** genome (Figs 1 and 3). Only one copy *Rpb2* sequence was obtained from accession PI 232151 and PI 372644, but two copies of *Pepc* sequences each from these accessions were obtained, and were phylogenetically grouped into **H1** and **H2**. Three copies of *Rpb2* and *Pepc* sequences were recovered from the accession PI 232147. The *Rpb2* sequence data indicated the presence of **StHH**, while *Pepc* data indicated the presence of **H1H1H2** sequences in this accession. Chloroplast data well separated the sequences of *E*. *trachycaulus* from **H**-genome species, indicating non-*Hordeum* species as maternal donor to *E*. *trachycaulus*, and presence of one copy of non-*Hordeum* genome in nuclear of tetraploid *E*. *trachycaulus*, most likely **St** genome as discussed above and suggested previously [41, 54–56].

In a study of tetraploid *Elymus canius* with **StH** genomes by [57], the *Rpb2* data also indicated presence of either **St1** or **St2** together with **H** genome in *E*. *caninus*. The GBSSI data indicated the presence of *Pseudoroegneria* (**St**), *Hordeum* (**H**) and an “unknown” *Pseudoroegneria*-like genome in *Elymus repens* [58]. Our *Rpb2* data here indicated that the **St** genome in *E*. *trachycaulus* was originated from either *P*. *strigosa*, *P*. *stipifolia*, *P*. *spicata* or *P*. *geniculate*. The *Hordeum*-like sequences of *E*. *trachycaulus* are polyphyletic in the *Pepc* tree, suggesting that the **H** genome in *E*. *trachycaulus* were contributed by multiple sources (Figs 1 and 3), whether due to multiple origins or to subsequent hybridization.

### Genome diversity and evolution

Allopolyploidization, brought about by inter-specific or inter-generic hybridization followed by chromosome doubling, contributes to the evolution of new functions in duplicated genes [59–61]. During or after the process of allopolyploidization, rapid sequence elimination and rearrangement, cytosine methylation, as well as transposable element activation and epigenetic gene silencing in allopolyploids might have been occurred [3– 6]. Rapid elimination of low-copy DNA gene from one genome is a general phenomenon in newly synthesized allopolyploids after hybridization or after chromosome doubling [7, 9]. The genome asymmetry caused by the lost of one parental gene copy was not restricted in *Triticum* or *Elymus* [8, 62], it was also evident in allotetraploid soybean [63–67].

It has cytological been confirmed that *E*. *trachycaulus* is allotetraploid [20, 21]. Two distinct copies of sequences for each single copy of nuclear gene are expected to be recovered from allotetraploid *E*. *trachycaulus*. However, two distinct copies were not recovered from all accessions for either *Rpb2* or *Pepc* gene. Only one copy *Rpb2* sequence was obtained from accession PI 232151 and PI372644, and one copy of *Pepc* sequence each was recovered from accession PI 440101, H10140 and PI 232150 even though more than ten clones were screened from each accession. Assuming no bias in cloning or PCR amplification, this gives a 99.9% chance of obtaining at least one copy of each of the two ancestral allelic types for the allotetraploid [68]. This might be due to mutation in the primers region causing failure of amplification of the “missing” gene copy. Another possibility might be genome convergent evolution in allopolyploids, partly because the **St** genome in *Elymus* species acquired this part of the sequence by the inter-genome introgression of sequence segments from the **H** genome to the **St** genome and abundant genome-wide recombination following the fusion of **St** and **H** gametes, accompanying the process of polyploidization. Genome-wide recombination between the **St** and **H** genomes could result in the two genome sequences at this location being identical to the extent that we could not distinguish one from the other in this specific DNA fragment [57]. There were growing evidences that homoeologous rearrangements in *Brassica napus* [69–73], and exchange among homoeologous chromosomes [74] might lead to genetic asymmetry expression and promote convergent evolution of the two parental genomes and phenotypic variation in newly formed polyploids.

Surprisingly, the *Pepc* phylogenetic tree showed that **St** copies were recovered from only 3 accessions (PI 387895, H10665A, and H3526), and other accessions had 2 to 3 different **H** copies except PI232150 and H10140, from which only one copy of **H** genome sequence was recovered (Figs 2 and 3). One scenario is that **St** copy might been missed and not be found, but this situation is less likely because most accessions did not show the **St** copy even though at least 10 clones screened, and it is less likely that the **St** copy from about 10 accessions missed at the same time.

Sequence alignment (Fig 2) revealed deletions and insertions between/among the different copies of **H** sequences from the same accession (Fig 2). It has been reported instability of the *Pepc* sequences within *Hordeum* as revealed by numerous insertions and deletions, with some of them involving gain or loss of Stowaway-like transposable elements [75]. The two copies of *Pepc* sequences each from accession H4228, PI 440098, and H3995 and PI 232147 which were phylogenetically grouped into the same clade might be caused by instability of *Pepc* sequences in **H**- genome. The two/three **H**-like sequences from accession PI 372644, PI 232151, PI 232147, PI 236685 were clearly separated into **H1** and **H2** clades in the phylogenetic analysis. The two distinct sequences each isolated from those accessions might be less likely explained by *Pepc* instabilities in *Hordeum* since phylogenetic analysis excluded the insertion/deletions. The two phylogenetic distinct copies of **H** sequences in these accessions might be caused by gene introgression from *Hordeum* into *E*. *trachycaulus* following polyploidization. Incomplete concerted evolution cannot be excluded which incompletely homogenized **St** copy of *Pepc* toward second **H** copy of *Pepc*. Concerted evolution appears to be a common feature of highly repetitive nuclear sequences, however, low-copy nuclear genes are also not free from concerted evolution [76, 77].
